# When he smiles: Attractiveness preferences for male faces expressing emotions

**DOI:** 10.1017/ehs.2023.28

**Published:** 2023-11-14

**Authors:** Mariana L. Carrito, Francisca Bismarck, Pedro Bem-Haja, David I. Perrett, Isabel M. Santos

**Affiliations:** 1 Centro de Psicologia da Universidade do Porto, Faculdade de Psicologia e de Ciências da Educação da Universidade do Porto, Rua Alfredo Allen, 4200-135 Porto, Portugal; 2 Centro de Investigação em Tecnologias e Serviços de Saúde, Departamento de Educação e Psicologia, Universidade de Aveiro, Aveiro, Portugal; 3 School of Psychology and Neuroscience, University of St Andrews, St Andrews, UK; 4 William James Center for Research, Departamento de Educação e Psicologia, Universidade de Aveiro, Aveiro, Portugal

**Keywords:** face attractiveness, sexual dimorphism, masculinity, emotional expression

## Abstract

The impact of sexual dimorphism on facial attractiveness has been controversial owing to contradictory results, particularly in studies on female preferences. Given that sexually dimorphic facial features, especially more masculine ones, have been previously related to the perception of anger, we investigated the bi-directional influence of emotional expressions and facial masculinity and explored their impact on women's preferences for facial masculinity. We confirmed the effect of facial sexual dimorphism on the perception of emotional cues (happiness and anger) and explored whether smiling or angry expressions influence women's perception of masculinity in male faces. Additionally, we examined women's preferences for emotionally expressive male faces altered along a continuum of masculinity. The results showed that masculinised faces are perceived as angrier, while feminised faces are perceived as happier (Experiment 1), and that angry faces are perceived as more masculine when compared with happy faces (Experiment 2). It is noteworthy that our Experiment 3 uncovered a pivotal finding: women prefer reduced feminisation in happy faces compared with neutral/angry faces. This suggests that the avoidance response observed towards masculinity is attenuated by a smiling expression. The current study introduces a new perspective to be considered when exploring the role of facial masculinity in women's attractiveness preferences.

## Graphical abstract

**Figure:**
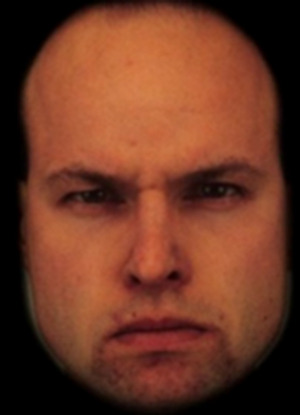

**Social media summary:** Emotional expression impacts women's preferences in male faces: smiling reduces avoidance response towards masculinity.

## 1. Introduction

According to evolutionary psychology theories, humans find certain characteristics attractive in faces because they signal mate quality (Little et al., 2011; Rhodes, 2006). Three factors have been identified that affect facial attractiveness: symmetry (Fink & Penton-Voak, 2002; Little et al., 2011; Mealey et al., 1999; Perrett et al., 1999; Rhodes, 2006), averageness (Fink & Penton-Voak, 2002; Little et al., 2011; Little & Hancock, 2002; Rhodes, 2006) and sexually dimorphic features (Fink & Penton-Voak, 2002; Johnston et al., 2001; Little et al., 2011; Little & Mannion, 2006; Rhodes, 2006).

Regarding the latter, the immunocompetence hypothesis suggests that humans may prefer mates with exaggerated secondary sexual characteristics because these features may signal strong immune system function and overall health. According to this hypothesis, men who have more masculine facial traits are more resilient to disease, owing to their ability to cope with the debilitating effects of higher levels of testosterone on their immune system (Fink & Penton-Voak, 2002; Foo et al., 2020) and hence should be perceived as more attractive by the opposite sex. While some studies exploring face shape preferences suggest that females do tend to prefer male faces that display more masculine traits (Holzleitner & Perrett, 2017; Johnston et al., 2001; Little & Mannion, 2006), others find that a feminised shaped male face is more agreeable to the female eye (Alharbi et al., 2020; Carrito et al., 2016, 2018; Little & Hancock, 2002; Perrett et al., 1998; Rhodes et al., 2003). Overall, the findings of studies on the attractiveness of male facial masculinity are inconsistent, and recent evidence has questioned the idea of masculinity being an honest signal of immune quality (e.g. Nowak et al., 2018; Jones et al., 2019; Jones et al., 2021).

Alternative justifications have been suggested for these inconsistent results. Some of the reasoning comes from cross-cultural studies that indicate women may adjust masculinity preferences according to environmental and economic demands. DeBruine et al. (2010a) suggested that women in environments with a high prevalence of pathogens and inaccessible or poor healthcare prefer masculine men. Scott et al. (2014) proposed an increased preference for masculine men in highly developed environments (with high urbanisation). More recent work highlights the positive relationship between women's preferences for masculine facial traits and countries’ sociosexuality (Alharbi et al., 2020; Marcinkowska et al., 2019).

Other explanations highlight the associations between facial masculinity and traits other than health or resistance to disease. One such association is the perception of dominance, which is positively associated with structural facial masculinity for judgments of both women (Johnston et al., 2001; Perrett et al., 1998; Puts, 2010; Swaddle & Reierson, 2002) and men (Muller & Mazur, 1997). In fact, the impact of masculine traits on the perception of dominance is more substantial and consistently positive compared with their effect on attractiveness (Puts et al., 2012). Boothroyd et al. (2007) showed facial masculinity to be associated with higher levels of perceived dominance, and lower perceptions of fidelity and commitment, suggesting that masculine men may be perceived as dominant and high status but also as unsuitable partners. Since more masculine faces are perceived as less warm, emotional, honest and cooperative, as well as having poorer quality as a parent, a preference for more feminine male faces may be an attempt to avoid negative behavioural attributes (Perrett et al., 1998).

There is another plausible, and probably compatible, explanation for the inconsistent results regarding the attractiveness of facial masculinity that relates to the perception of emotion. The perception of emotional expressions is a prominent factor in human facial attractiveness, especially because emotional expressions capture our eye effectively, with evidence that even newborns can discriminate, and show preference for, positive facial expressions (Farroni et al., 2007). Evidence shows that perception of others’ emotional state happens automatically (Todorov et al., 2009) and is based on subtle cues present even in apparently neutral faces (a phenomenon known as emotional-face overgeneralisation; Zebrowitz, 2017). Neutral expression faces may easily be perceived as happy when appearing trustworthy, while low trustworthiness cues make neutral faces appear angrier (Oosterhof & Todorov, 2008). Said et al. (2009) showed that when neutral faces are perceived as having a positive valence, they tend to resemble happiness, while those perceived as negative tend to resemble expressions of disgust and fear. Additionally, faces that convey a sense of threat tend to bear a resemblance to expressions of anger.

Also, it is now understood that the perception of emotional expressions does not happen independently from the processing of facial sex cues (Atkinson et al., 2005; Becker, 2017). Previous research has explored the influence of manipulating facial structure on the interaction between sex and emotion perception, proposing that there is a structural overlap between sex and emotional facial features (Craig & Lee, 2020). For example, the results from seven experiments by Becker et al. (2007) indicated that participants were quicker and more accurate in identifying an angry facial expression when portrayed by a male, and a happy facial expression when portrayed by a female. With the manipulation of androgynous neutral faces, the authors found that lowering the brow ridge made participants perceive these faces as both more masculine and angrier (Becker et al., 2007). In line with this perspective, Sell et al. (2014) proposed a functional explanation for the evolution of angry facial expressions in men, suggesting that these expressions serve as honest signals of male formidability by modulating the specific muscular movements associated with anger. Male formidability can be communicated in structural facial traits such as bizygomatic width, which was proven to be highly sexually dimorphic in multivariate analyses when adjusting for allometry (Caton & Dixson, 2022).

Subsequent studies revealed that participants demonstrate faster response times and/or higher accuracy when categorising anger expressions on male faces compared with female faces (e.g. Aguado et al., 2009; Le Gal & Bruce, 2002), showing that there is a resemblance between masculine facial traits and the facial expression of anger (see also Adams et al., 2012). Hess et al. (2009) also found that androgynous faces expressing anger are more often identified as being male than female, offering additional evidence of the perceptual overlap between facial masculinity and the expression of anger. Such anger signalling might not be appealing in mating contexts, by inducing avoidance instead of approach. Consequently, we can hypothesise that female participants in previous attractiveness studies could have perceived more masculine (or masculinised) male faces as angrier faces, assessing them as more unpleasant and unattractive.

The goal of our study was to improve the understanding of how emotional expressions influence the preferences of heterosexual women for face masculinity. To do so, we first conducted Experiment 1 with the purpose of exploring whether sexually dimorphic differences in the morphology of neutral faces influence the perception of emotion. We hypothesised that more masculine faces would be perceived as angrier, and more feminine faces would be perceived as happier. In Experiment 2, we explored whether facial expression influences the perception of masculinity in men. We hypothesised that angry faces would be perceived as more masculine, and that happy faces would be perceived as more feminine. Finally, in Experiment 3, we tested whether showing faces expressing various emotions would lead to different masculinity preferences. Female participants were asked to manipulate images of male faces with angry, neutral and happy expressions, increasing or decreasing the level of masculinisation until the faces looked the most attractive. We hypothesised that participants would prefer higher masculinisation levels (or lower femininisation levels) in happy faces when compared with neutral or angry faces, since a happy expression should counteract the perception of anger in more masculine faces – therefore making the masculine face shape more attractive.

## 2. Experiment 1

The aim of this experiment was to explore whether sexually dimorphic differences in face morphology elicit the perception of different emotional states in neutral faces. We anticipated that participants would perceive neutral male faces as being angrier than neutral female faces and neutral female faces as being happier than neutral male faces. We also expected that, overall, masculinised neutral faces would be perceived as angrier than feminised ones, and feminised neutral faces would be perceived as happier than masculinised ones. Consequently, masculinised neutral male faces were expected to be perceived as the angriest between our four conditions and feminised neutral female faces as the happiest.

### 2.1. Method

#### 2.1.1. Participants

We used G*Power 3.1.9.7 software to estimate sample size, considering a medium effect size (*F* = 0.25), an alpha of 0.05 and a power of 0.8, which indicated a minimum total sample size of 64 participants. Thirty-six men (*M*_age_ = 25.53, SD = 4.79) and 39 women (*M*_age_ = 27.80, SD = 4.20) participated in this experiment. Thirty-seven participants (19 women) were randomly assigned to the anger condition and 40 participants (22 women) were randomly assigned to the happy condition. Participants considered themselves to be averagely attractive (median = 4.00) when responding to the self-attractiveness scale, 75.3% (*N* = 58) reported being in a relationship at the time of the experiment and 51.2% of women (*N* = 21) reported using hormonal contraceptives. All participants were Caucasian, identified as heterosexual and reported good or corrected vision.

#### 2.1.2. Stimuli

Twenty-four composite faces (12 male faces and 12 female faces) were created using photographs from a St Andrews database of Caucasian adult faces. Each of the faces was an average of three individual neutral faces from different individuals (see Carrito et al. 2016 for detailed procedures). The faces were delineated with 192 points (with *x* and *y* coordinates) using Psychomorph software (Tiddeman et al., 2001). Delineation allowed face manipulation based on the shape difference between two endpoint shape masks, resulting in a feminised version (–100%) and a masculinised version (+100%) of each original composite face. The hair, neck, ears and background were cleared from the image to ensure that they would not affect the results, as suggested in DeBruine et al. (2010b). Two sets with six feminised and six masculinised versions of faces of each sex were created so that only one of the versions of each individual would be presented to the participants.

#### 2.1.3. Procedure

The authors assert that all procedures contributing to this work comply with the ethical standards of the relevant national and institutional committees on human experimentation and with the Helsinki Declaration of 1975, as revised in 2008. The procedures of the current experiment were approved by the Ethics Committee of the Faculty of Psychology and Educational Sciences of the University of Porto.

The task was built on the online platform Qualtrics (https://www.qualtrics.com/). Both sex participants started by signing an informed consent form and were then asked to answer a basic demographic questionnaire. Through this questionnaire, we intended to assess variables such as age, gender, self-rated attractiveness (measured on a seven-point scale, where 1 meant very unattractive and 7 meant very attractive), relationship status and hormonal contraceptive use. In addition to these factors, we asked about the participant's vision with the purpose of excluding people with uncorrected vision problems. Moreover, volunteers were selected to partake in the study only if they identified as Caucasian and heterosexual, between the ages of 18 and 35. In the experimental task, participants were asked to evaluate neutral faces of each sex in terms of perceived facial expression (anger or happiness). Twenty-four faces of each sex were presented to each participant along with a 100-point visual analogue scale ranging from (1) Not angry to Angry or (2) Not happy to Happy. Two blocks of faces were presented, with masculinised/feminised versions of each face identity appearing in only one of them. In each block, there were 12 male faces and 12 female faces. The order of presentation of the blocks was randomised. Participants randomly assigned to the anger condition were asked to rate the emotional expression of each face presented, ranging from ‘Not angry at all’ to ‘Angry’. Participants randomly assigned to the happy condition had to rate the faces presented using the anchor points ‘Not happy at all’ to ‘Happy’. The discrete value selected was not shown to the participant.

#### 2.1.4. Data analysis

All statistical analyses were performed using SPSS 26.0.0.0 and the significance level was set at *p* = 0.050. The mean degree of perceived emotion by each participant in each condition (male-feminised, male-masculinised, female-feminised, female-masculinised) was calculated. Interclass correlation coefficients calculation showed a high to moderate degree of consistency among stimuli of each category (ICC_male-feminised_ = 0.648, 95% CI [0.543, 0.739]; ICC_male-masculinised_ = 0.842, 95% CI [0.795, 0.883]; ICC_female-feminised_ = 0.918, 95% CI [0.894, 0.939]; ICC_female-masculinised_ = 0.798, 95% CI [0.738, 0.850]). Therefore we used scores aggregated across stimuli in each category for analysis. Shapiro–Wilk tests showed that the residuals for the conditions male-masculinised and male-feminised had non-normal distributions. Hence, non-parametric tests were conducted. Matched pairs rank-biserial correlations (‘mpRBC’) were calculated as effect size measures for Wilcoxon signed rank tests.

### 2.2. Results

#### 2.2.1. Anger evaluation

**Figure 1.**
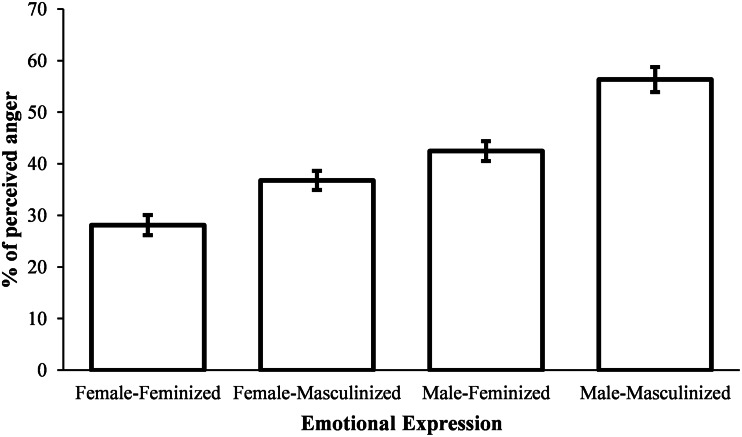
Mean level of perceived anger for each condition in Experiment 1. Error bars show standard errors of the mean.

To analyse the effect of sexually dimorphic facial traits on the level of perceived anger for each sex, we performed two Wilcoxon signed ranks tests with Bonferroni corrected alpha of 0.025. These comparisons turned out to be significant in both cases, showing that masculinised male faces were associated with increased anger evaluations compared with feminised male faces, *Z* = –5.30, *p* < 0.001, VS-MPR = 194226, mpRBC = –1, as were masculinised female faces when compared with feminised female faces, Z = −4.59, *p* < 0.001, VS-MPR = 78060, mpRBC = –0.886, 95% CI for Rank-Biserial Correlation [–0.934, –0.739] (see Figure 1). Male faces in general were also perceived as angrier when compared with female faces, *Z* = −5.30, *p* < 0.001, VS-MPR = 1.01 × 10^9^, mpRBC = –1.

Regarding differences between male and female participants, Mann–Whitney tests showed that groups did not differ on how they perceived all four categories of faces (all *p*-values > 0.111).

#### 2.2.2. Happiness evaluation

**Figure 2.**
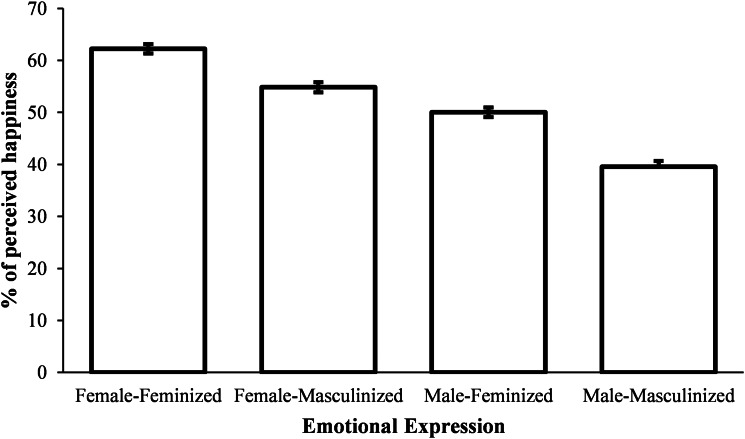
Mean level of perceived happiness for each condition in Experiment 1. Error bars show standard errors of the mean.

When exploring the effect of sexually dimorphic traits in the group that performed the happiness evaluation, Wilcoxon signed ranks tests with Bonferroni corrected alpha of 0.025 also showed statistically significant differences. Masculinised male faces were associated with lower happiness evaluations compared with feminised male faces, *Z* = −5.48, *p* < 0.001, VS-MPR = 347705, mpRBC = 0.995, 95% CI for Rank-Biserial Correlation [0.989, 0.998], as did masculinised female faces when compared with feminised female faces, *Z* = −4.97, *p* < 0.001, VS-MPR = 24894, mpRBC = 0.897, 95% CI for Rank-Biserial Correlation [0.800, 0.949] (see Figure 2). Female faces in general were perceived as happier than male faces, *Z* = −5.51, *p* < 0.001, VS-MPR = 402678, mpRBC = –1.

Once again Mann–Whitney tests showed that male and female participants did not differ on how they perceived all four categories of faces (all p-values > 0.229).

## 3. Experiment 2

The aim of this experiment was to do the reverse of Experiment 1 and explore whether facial expression influences the perception of masculinity, particularly in male faces. We anticipated that angry faces would be perceived as more masculine when compared with the happy and neutral conditions, and that happy faces would be perceived as more feminine when compared with the angry and neutral conditions.

### 3.1. Method

#### 3.1.1. Participants

Sample size was estimated using G*Power 3.1.9.7 software, considering a medium effect size (*F* = 0.25), an alpha of 0.05 and a power of 0.8. A total sample of 32 participants was obtained. We collected online data from 35 Caucasian heterosexual women, between the ages of 18 and 35 years old (*M*_age_ = 22.80, SD = 4.21), with normal or corrected vision. These women considered themselves to be averagely attractive (median = 4.00), 71.4% (*N* = 25) reported being in a relationship at the time of the experiment, and 62.9% (*N* = 22) reported using hormonal contraceptives.

#### 3.1.2. Stimuli

Ninety photographed faces from 30 individuals showing three different emotions (angry, neutral and happy) from the Karolinska Directed Emotional Faces (Lundqvist et al., 1998) were used. Each of the 90 faces was delineated with 192 points (using Psychomorph software as in Experiment 1) to delimit the face areas that would be transformed. Three levels of masculinisation for each emotional face were obtained: –50% (50% feminisation), 0% (no change), and +50% masculinisation, considering the shape difference between the average male and average female face shapes of DeBruine and Jones (2015). A smaller range of masculinisation was considered in this experiment since changes above 50% cause significant distortions in faces expressing emotions. Again, the hair, neck, ears and background were occluded from view. We obtained 270 images that corresponded to the sum of all nine variations (in expression and masculinity) of each of the 30 individuals mentioned above. To avoid long-lasting sessions that could result in participants’ fatigue and low performance, the 270 faces were divided into three sets so that participants would be presented with a maximum of 90 faces.

#### 3.1.3. Procedure

**Figure 3.**
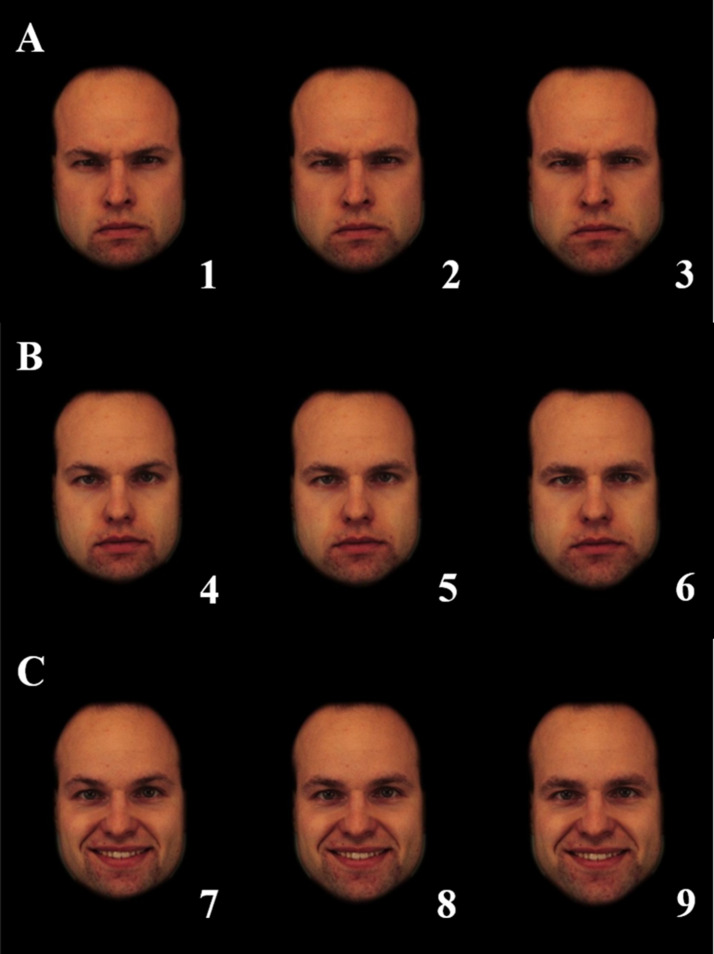
Examples of the images presented in Experiment 2 by emotion (A, angry; B, neutral; C, happy) and by masculinization level (1, 4, 7 – 50% feminized; 2, 5, 8 – original; 3, 6, 9 – 50% masculinized).

Like Experiment 1, the procedure was approved by the Ethics Committee of the Faculty of Psychology and Educational Sciences of the University of Porto and the task was built on the online platform Qualtrics. Participants started by signing an informed consent form and answered a basic demographic questionnaire, similar to the one presented in Experiment 1. Later, participants were presented with 90 faces (10 individuals × 3 emotions × 3 masculinisation levels). The selected set of 90 faces and the order of presentation of the faces were randomised (although faces from the same individual were never shown following each other). Therefore, each participant was asked to rate pictures representing nine possible combinations of emotional expression and sexual dimorphism (1, angry feminised; 2, angry original; 3, angry masculinised; 4, neutral feminised; 5, neutral original; 6, neutral masculinised; 7, happy feminised; 8, happy original; 9, happy masculinised). Participants were asked to rate how masculine the male face appeared to them, on a visual scale from 0 (*extremely feminine*) to 100 (*extremely masculine*) (see Figure 3).

#### 3.1.4. Data analysis

The mean degree of masculinity perceived in each condition (angry-feminised, angry-original, angry-masculinised, happy-feminised, happy-original, happy-masculinised, neutral-feminised, neutral-original and neutral-masculinised) was calculated for each participant. Mean values of interclass correlation coefficients show a high to moderate degree of consistency among stimuli of each category (ICC_angry-feminised_ = 0.752, 95% CI [0.493, 0.915]; ICC_angry-original_ = 0.812, 95% CI [0.616, 0.935]; ICC_angry-masculinised_ = 0.792, 95% CI [0.574, 0.929]; ICC_happy-feminised_ = 0.868, 95% CI [0.734, 0.952]; ICC_happy-original_ = 0.867, 95% CI [0.730, 0.935]; ICC_happy-masculinised_ = 0.893, 95% CI [0.783, 0.962]; ICC_neutral-feminised_ = 0.620, 95% CI [0.222, 0.869]; ICC_neutral-original_ = 0.589, 95% CI [0.157, 0.860]; ICC_neutral-masculinised_ = 0.797, 95% CI [0.586, 0.929]). Therefore we used scores aggregated across stimuli in each category for analysis. Shapiro–Wilk tests confirmed the normality of the residuals for all the conditions, hence parametric tests were applied.

### 3.2. Results

**Figure 4.**
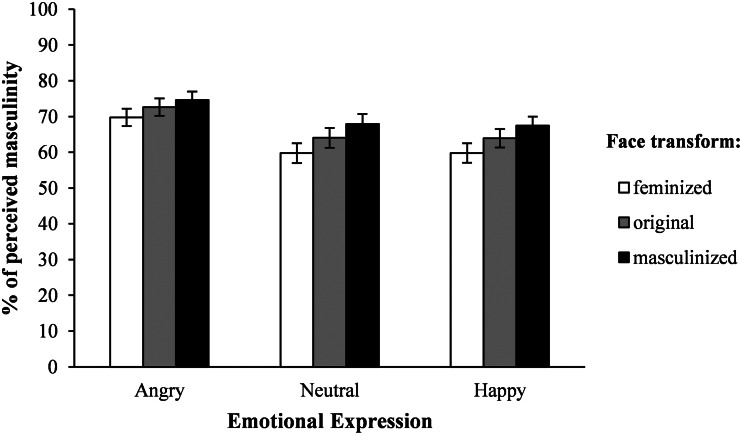
Mean level of perceived masculinity for each condition in Experiment 2. Error bars show standard errors of the mean.

A two-way repeated measures ANOVA (within-subjects factors: emotional expression and level of masculinisation) revealed a significant main effect of emotional expression, *F* (1.64, 55.65) = 31.55, *p* < 0.001, *η_p_*^2^ = 0.481, indicating that angry faces were perceived as more masculine (*M* = 72.33, SE = 2.37) when compared with happy (*M* = 63.91, SE = 2.74) and neutral (*M* = 63.71, SE = 2.56) faces (see Figure 4). The main effect of level of masculinisation was, as expected, statistically significant, *F* (1.70, 57.72) = 66.84, *p* < 0.001, *η_p_*^2^ = 0.663, as masculinised faces (*M* = 69.99, SE = 2.42) were perceived as more masculine compared with original versions (*M* = 66.87, SE = 2.49) which in turn were also perceived as more masculine compared with feminised ones (*M* = 63.09, SE = 2.54). Moreover, there was a significant interaction between the factors, *F* (4, 136) = 3,46, *p* = 0.01, *η_p_*^2^ = 0.092, showing that for angry expressions, all faces were considered to be highly masculine, whereas for neutral and happy faces, increased levels of masculinisation were related to an increase in perceived masculinity (all *p_s_* ≤ 0.006).

## 4. Experiment 3

The aim of this experiment was to explore female preferences for masculinity in faces expressing different emotional expressions. We anticipated that participants would prefer increased levels of masculinity in happy faces when compared with the neutral or angry versions of such faces, since happy faces were associated with increased perceived femininity (or lower masculinity) in the previous experiment.

### 4.1. Method

#### 4.1.1. Participants

Once again, the sample size was estimated using G*Power 3.1.9.7 software, considering a medium effect size (*F* = 0.25), an alpha of 0.05 and a power of 0.8. A total sample of 78 participants was obtained. Exclusion criteria focused on the required characteristics of gender (we only accepted people who identify as women), ethnicity (Caucasian), sexual orientation (heterosexual) and age (18–35 years old). Eighty-four heterosexual women, between the ages of 18 and 34 years old (*M* = 22.77, SD = 4.12) participated in this experiment. Six participants were later eliminated from the analyses owing to responding in less than a second in more than a third of the trials which was interpreted as a careless and random participation. The remaining 78 participants (*M*_age_ = 22.87, SD = 4.22) were considered in the analyses. These female participants considered themselves to be averagely attractive (median = 4.00), 41% (*N* = 32), reported being in a relationship at the time of the experiment, and 53.8% (*N* = 42) reported using hormonal contraceptives.

#### 4.1.2. Stimuli

From the 30 faces used in Experiment 2, we chose the 10 male faces and their three respective emotional expressions with the highest accuracy ratings in the masculinisation judgement task to include in this experiment – images that scored higher in perceived masculinity when masculinised and scored lower in perceived masculinity when feminised. The manipulation of the 30 male KDEF faces (Lundqvist et al., 1998) was done through Psychomorph (Tiddeman et al., 2001), as previously described in Experiment 2. A continuum of 11 images was created for each of the 30 selected faces, ranging from –50% masculinised to +50% masculinised in face shape. Once again, the hair, neck, ears and background were cleared from the image to ensure that they would not influence the results.

#### 4.1.3. Procedure

The procedure of the current experiment was approved by the Ethics Committee of the Faculty of Psychology and Educational Sciences of the University of Porto and by the University Teaching and Research Ethics Committee of the University of St Andrews. Volunteers were given information about the study and had to give consent before initiating the task, and were then asked to answer a demographic questionnaire, similar to the one presented in the previous two experiments.

The experiment consisted of an online interactive task, programmed in php and html languages, in which the participants changed the level of masculinity of the images. While unaware of the nature of the face manipulation, participants were asked to change the presented faces, searching for the most attractive appearance in each. Thirty images were shown (10 faces × 3 emotions), in random order, which could be altered by a horizontal mouse movement. Mouse movement resulted in a more feminine or more masculine face shape (within a –50% to +50% masculinity range), a procedure also adopted in Carrito et al. (2016). All participants were presented with the same faces, portraying the same emotional expressions: 10 angry, 10 happy and 10 neutral faces.

#### 4.1.4. Data analysis

We calculated the mean degree of masculinity preferred by each participant when manipulating neutral, happy and angry faces. A moderate degree of consistency among stimuli of each category was found (ICC_neutral_ = 0.725, 95% CI [0.643, 0.796]; ICC_happy_ = 0.635, 95% CI [0.526, 0.730]; ICC_angry_ = 0.695, 95% CI [0.604, 0.774]). Therefore we used scores aggregated across stimuli in each category for analysis. Shapiro–Wilk tests confirmed the normality of the residuals for all the conditions except for neutral faces which, nonetheless, showed acceptable skewness of 0.85 (SE = 0.27) and kurtosis of 1.12 (SE = 0.56). Thus, parametric tests were considered to explore levels of preferred masculinity between conditions.

### 4.2. Results

A significant preference for femininity was found in all conditions: angry (*M* = –15.56, SD = 14.50, one sample *t*-test against no change in masculinity, *t* (77) = –9.48, *p* < 0.001, *d* = 1.073), neutral (*M* = –16.10, SD = 14.60, one sample *t*-test against no change in masculinity, *t* (77) = –9.74, *p* < 0.001, *d* = 1.103), or happy (*M* = –11.50, SD = 13.68, one sample *t*-test against no change in masculinity, *t* (77) = –7.43, *p* < 0.001, *d* = 0.841).

**Figure 5.**
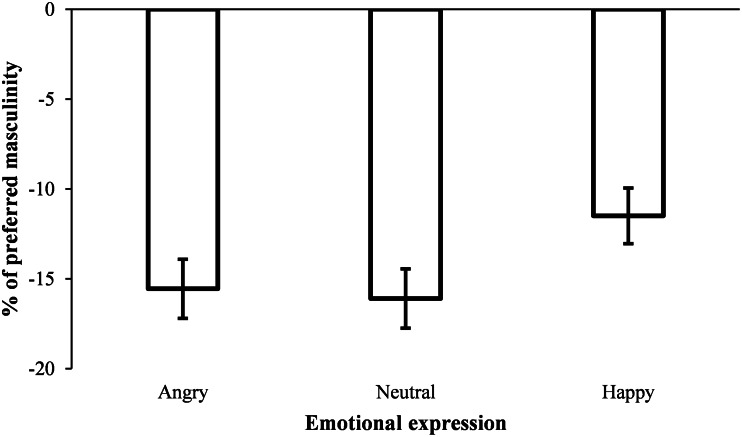
Mean masculinity level preferred according to the emotional expression of the face stimuli in Experiment 3. Error bars show standard errors of the mean.

A one-way repeated measures ANOVA (within-subjects factor: emotional expression) revealed a significant effect of emotional expression, *F* (2, 154) = 6.58, *p* = 0.002, *η_p_*^2^ = 0.79. Post hoc comparisons using Bonferroni adjustment indicated that the preference for femininity was lower in happy faces when compared with neutral faces (*p* = 0.002) and angry faces (*p* = 0.027) (see Figure 5). The other comparisons were not statistically significant.

## 5. Discussion

With this set of experiments, our aim was to understand the role of emotional expression in the preferences of heterosexual women for sexually dimorphic features in male faces. To do so, we evaluated the interdependence between the perception of masculinity and emotional expression in Experiments 1 and 2, and proceeded to explore our main hypothesis further in Experiment 3.

In Experiment 1 we evaluated whether sexually dimorphic differences in face morphology elicit the perception of different emotional states. Similarly to previous research (Adams et al., 2012; Becker et al., 2007), the results showed that male faces, in general, were perceived as angrier than female faces, and that female faces were, in general, perceived as happier than male faces. Moreover, we have found that masculinised faces were perceived as angrier than feminised faces, as well as feminised faces being perceived as happier than masculinised faces. These later results are concordant with a recent study showing that facial width-to-height ratio, a putative sexually dimorphic face structure in young adults (Summersby et al., 2022), influences the perception of anger and happiness in neutral faces (Deska et al., 2018). In Experiment 2, we explored whether facial expression influences the perception of masculinity in male faces, in the eyes of female participants. We hypothesised that angry faces would be perceived as more masculine and happy faces as more feminine. Results confirmed that angry faces were rated as more masculine than happy or neutral faces.

Our results from ratings in Experiments 1 and 2 concur with the conclusions of Becker et al. (2007), Hess et al. (2009) and others that focused analyses on reaction time (e.g. Aguado et al., 2009; Le Gal & Bruce, 2002; Smith et al., 2017). Both types of study suggest that there is a perceptual connection between sexual dimorphism in facial structure and emotional expressions. Perception of female faces seems to be biased towards the perception of happiness and the perception of male faces towards the perception of anger. The reason for this seems to lie on the morphologic similarities between the expression of anger and the sexually dimorphic structure of male faces (i.e. lower eyebrows and a smaller gap between eyebrows).

Crucially, in Experiment 3, we tested whether showing male faces expressing happiness to female participants would lead to different masculinity preferences compared with when we presented neutral or angry male faces. Our hypothesis was that females would prefer higher levels of masculinisation when presented with happy faces, since the expression of happiness was expected to neutralise the perception of anger in more masculine faces. Therefore, it was expected that participants would prefer a more feminine appearance as an avoidance response to the anger in male faces portraying neutral or angry expressions. The avoidance response in this context could be due to a general self-preservation behaviour, or a female mate-choice strategy (Borras-Guevara et al., 2017). Marsh et al. (2005) showed that anger expressions facilitate avoidance-related behaviour, suggesting this expression is generally aversive. On the other hand, McDonald et al. (2015: 438), suggested that ‘women may be equipped with a threat-management system that functions to protect reproductive choice by avoiding individuals that may have historically posed an increased threat of sexual coercion’. Since the expression of anger is likely to be perceived as threatening, it would make sense that an avoidance response would take place to defend reproductive choice (McDonald, et al., 2015). Threat perceptions have been shown to be increased by sexually dimorphic facial cues, namely facial hair (Craig et al., 2019; Dixson & Vasey, 2012; Dixson et al., 2021). Accordingly, our results showed that participants chose to feminise the shape of faces in general, although the preference for feminisation was significantly lower for happy faces when compared with angry or neutral faces.

These findings are important because they show that a changeable trait, namely emotional expression, is sufficient to impact attraction to masculinity. By showing that women's preferences regarding sexual dimorphism change when a face smiles, this study adds complexity to the value of sexual dimorphism in mate choice. We propose that preferences for more feminised faces are an attempt to ‘soften’ the negative attributions, such as apparent hostility, associated with the subtle (or frank) expression of anger. This interpretation is in line with conceptualisations of sexual dimorphism as a dominance marker and a by-product of intra-sexual competition (Puts, 2010; Puts et al., 2012), but further research is required to explore the relationship between anger, dominance and masculinity face preferences. Moreover, another important step following this study would be to test male preferences regarding female expressions and their interaction with femininity. It would have been interesting to investigate whether the advantage of perceiving anger in masculinised faces exhibits sex-specific patterns, as proposed by Becker et al. (2007).

Finally, it could be valuable to replicate the reported attractiveness findings through alternative experimental approaches. Previous studies on facial attractiveness judgments have demonstrated variations in results depending on the methodology employed. For instance, when comparing the two-alternative forced-choice method to subjective ratings of attractiveness and objective measurements of facial shape characteristics, differences in outcomes have been observed (Jones & Jaeger, 2019; Lee et al., 2021; Scott et al., 2010). In a more recent study by Dong et al. (2023), questions were raised about the predictive value of masculine shape characteristics and their role in determining dominance, as different perceptions of dominance were obtained based on the methodological approach used to assess shape-manipulated images.

## 6. Conclusion

To our knowledge, this study is the first to explore the relationship between the preferences of women for the sexually dimorphic shape of male faces and the perception of emotional expressions. Based on the three experiments, we conclude that emotional expression does impact women's preferences for male masculinity. The extent of the interaction between expression and masculinity is unknown, but differences in mouth curvature and hence apparent resting expression may contribute to the divergence in the results of studies on attraction to masculinity.

## Data Availability

The data that support the findings of this study are openly available in Open Science Framework at https://osf.io/q6gpc/?view_only=eb3eab41420842d398d46a46c81b3614.
